# Adalimumab Biosimilar GP2017 versus Adalimumab Originator in Treating Patients with Inflammatory Bowel Diseases: A Real-Life, Multicenter, Observational Study

**DOI:** 10.3390/biomedicines10081799

**Published:** 2022-07-26

**Authors:** Giammarco Mocci, Giorgia Bodini, Leonardo Allegretta, Alessia Immacolata Cazzato, Stefania Chiri, Giovanni Aragona, Patrizia Perazzo, Antonio Ferronato, Maria Giovanna Graziani, Cristiano Pagnini, Costantino Zampaletta, Camilla Graziosi, Marcello Picchio, Walter Elisei, Giovanni Maconi, Antonio Tursi

**Affiliations:** 1 Division of Gastroenterology, “Brotzu” Hospital, 09124 Cagliari, Italy; giammarco.mocci@gmail.com; 2 Department of Internal Medicine and Medical Specialties, Division of Gastroenterology, IRCCS “San Martino” Hospital, University of Genoa, 16132 Genoa, Italy; giorgia.bodini@unige.it; 3 Division of Gastroenterology, “Santa Caterina Novella” Hospital, 73050 Galatina, Italy; leonardo.allegretta@tin.it (L.A.); alessiacazzato@gmail.com (A.I.C.); stefania.chiri@gmail.com (S.C.); 4 Division of Gastroenterology, “Guglielmo da Saliceto" Hospital, 29100 Piacenza, Italy; g.aragona@ausl.pc.it (G.A.); p.perazzo@ausl.pc.it (P.P.); 5 Digestive Endoscopy Unit, ULSS7 Pedemontana, 36061 Santorso, Italy; antonio.ferronato@aulss7.veneto.it; 6 Division of Gastroenterology, “S. Giovanni-Addolorata” Hospital, 00184 Rome, Italy; mg.grazianio@virgilio.it (M.G.G.); cpagnini@hsangiovanni.roma.it (C.P.); 7 Division of Gastroenterology, “Belcolle” Hospital, 92911 Viterbo, Italy; zcosta@libero.it (C.Z.); camilla.graziosi@gmail.com (C.G.); 8 Division of General Surgery, “P. Colombo” Hospital, ASL Roma 6, 00041 Rome, Italy; marcello.picchio.63@alice.it; 9 Division of Gastroenterology, “S. Camillo” Hospital, 00152 Rome, Italy; walter_elisei@hotmail.com; 10 Division of Gastroenterology, “L. Sacco” University Hospital, 20157 Milan, Italy; giovanni.maconi@unimi.it; 11 Territorial Gastroenterology Service, ASL BAT, 76123 Andria, Italy; 12 Department of Medical and Surgical Sciences, Post-Graduate School of Digestive Diseases, Catholic University, 20123 Rome, Italy

**Keywords:** adalimumab, biosimilar, GP2017, inflammatory bowel disease

## Abstract

The approval of adalimumab (ADA) biosimilars for inflammatory bowel disease (IBD) has reduced the cost of treatment. While several ADA biosimilars are currently available, comparative data on the ADA biosimilar GP2017 (Hyrimoz^TM^) and its originator (Humira^TM^) in IBD are lacking. We compared the efficacy and safety of GP2017 versus originator in IBD outpatients in an Italian real-life setting. This retrospective analysis enrolled consecutive IBD patients with complete clinical, laboratory, and endoscopic data. Clinical activity was assessed with the Mayo score in ulcerative colitis (UC) and the Harvey–Bradshaw Index in Crohn’s disease (CD). The primary endpoints were the induction of remission and the safety of GP2017 versus ADA originator. One hundred and thirty-four patients (30.6% with UC and 69.4% with CD, median age 38 years) were enrolled: 62 (46.3%) patients were treated with GP2017, and 72 (53.7%) with ADA originator; 118 (88.1%) patients were naïve to ADA. Clinical remission was obtained in 105 (78.4%) patients, during a median follow-up of 12 months, 82.3% and 75% in the GP2017 and ADA originator groups, respectively (*p* = 0.311). Treatment was well tolerated in both groups. This analysis of real-world data suggests that GP2017 and its originator are equivalent in terms of efficacy and safety in patients with IBD.

## 1. Introduction

Inflammatory bowel diseases (IBD), which primarily include Crohn’s disease (CD) and ulcerative colitis (UC), are frequently observed in the Western world, with 6.8 million cases of IBD reported globally in 2017 [1]. The occurrence of IBD involves a complex interaction between genetic and environmental factors [2]. In addition, due to the relapsing and remitting nature of their disease course, aggressive treatment of CD and UC is often required to prevent further complications [3]. 

The pleiotropic proinflammatory cytokine tumor necrosis factor α (TNFα), performs a crucial role in the pathogenesis of CD and UC, and anti-TNFα agents were the first biological drugs approved to treat IBD [3]. However, the cost of TNFα inhibitors is significantly higher than traditional treatments [4,5]. An increasing number of biosimilars have been developed following expiration of the original biologic patent, and their efficacy and safety are estimated to be the same as their originators [6].

Adalimumab (ADA), a fully human monoclonal antibody targeting TNF both in its soluble and membrane-bound form, is effective and safe for the long-term management of IBD over a median follow-up of 60 months [7]. In Italy, ADA biosimilars are now approved to treat IBD. However, at present, real-life data on the efficacy and safety of the ADA biosimilars ABP501 (Amgevita^TM^, Amgen Inc., Thousand Oaks, CA, USA), SB5 (Imraldi^TM^, Samsung Bioepis UK Limited, Brentford, UK), MSB11022 (Idacio^®^, Fresenius Kabi, Toronto, ON, Canada), and GP2017 (Hyrimoz^TM^, Sandoz GmbH, Holzkirchen, Germany) are currently limited [8,9,10,11]. 

The ADA biosimilar GP2017, was approved in January 2019 in Italy to treat patients with IBD [12]. Importantly, comparable efficacy and safety outcomes between GP2017 and three other biosimilars (SB5, APB501, and MSB11022) were identified in an Italian real-life observational study in IBD patients [11]. However, data comparing GP2017 and its ADA originator (Humira^TM^) in IBD patients are lacking. We compared the efficacy and safety of GP2017 and ADA originator in treating IBD outpatients from nine IBD centers in a real-life Italian setting.

## 2. Materials and Methods

This was a retrospective, multicenter study, conducted in nine Italian IBD centers (four in the north of Italy—Milan, Genoa, Piacenza, Vicenza—, two in the center—Rome and Viterbo—, two in the south—Andria, Lecce— and one in Sardinia—Cagliari), enrolling consecutive IBD outpatients with UC or CD treated with GP2017 due to their unresponsiveness to standard treatments. 

Patients included had completed at least the induction treatment between 1 January 2020, and 31 May 2021. As a control group, data were also collected from patients treated with ADA originator. Due to the introduction of GP2017 to the Italian market in 2019, we limited the control group to patients who had a follow-up of no longer than one year. 

Eligible patients included men and women (>18 years), diagnosed with UC or CD following standard endoscopic, radiology, and/or histological criteria [2]. Patients were not considered in case their medical record reported either an active hepatitis B virus or tuberculosis infection. No patients with indeterminate colitis were found among this population.

Written informed consent was obtained from all the patients before undergoing endoscopy and ADA treatment, and all patient data were anonymous. The study was conducted following clinical practice guidelines and in accordance with the ethical standards established by the Declaration of Helsinki. Due to its retrospective design, no Ethic Committee approval was required by the Italian law. Indeed, according to the Italian law, a formal patient consent, as well as Ethic Committee approval, is not required for this type of study [13,14,15]. This study was notified to the Ethic Committee of “Brotzu” Hospital on 28 April 2021, with the protocol number PROT PG/2021/10115.

### 2.1. Study Treatment

For patients new to ADA, both GP2017 and originator were administered subcutaneously at the following doses: 160 mg at week 0, 80 mg at week 2, and then 40 mg every 2 weeks to maintain remission. For patients previously treated with ADA originator who switched to GP2017, a dose of 40 mg was administered subcutaneously every 2 weeks to maintain remission after switch. 

The need for treatment discontinuation and/or dose escalation, or the addition of concomitant medications, such as oral and topical aminosalicylates, steroids, and immunosuppressants, were determined by the treating physician.

### 2.2. Clinical Assessment

Demographic and clinical data of the patients were collected through a shared database. At baseline the following information were collected: sex, age at diagnosis, smoking status, disease extension and duration, previous immunosuppressive and biologic therapies (anti-TNFα or anti-integrin), concomitant medications, C-reactive protein (CRP) and fecal calprotectin (FC) levels, Mayo score and Mayo subscore for endoscopy for UC patients, and Harvey–Bradshaw index (HBI) for CD patients. Patients who were naïve to ADA were clinically assessed at baseline, 2, 3 and 6 months, and every 6 months thereafter during follow-up; patients who switched from ADA originator to GP2017 for nonmedical reasons were assessed every 6 months. 

The Montreal classification was used to define the extent of the disease [16]. Disease severity was determined using the Mayo score [17] or the HBI [18] in UC and CD patients, respectively. All included patients naïve to ADA treatment presented with active disease defined as a Mayo score of ≥3 points for UC patients and as an HBI score of ≥5 points for CD patients, despite concomitant therapy. 

### 2.3. Endoscopy

All patients new to ADA underwent an ileocolonoscopy before the start of biologic treatment, according to the standard protocol in the participating centers. The same procedure was followed also before switching to GP2017. In CD patients with an upper gastrointestinal location, esophagogastroduodenoscopy and ileocolonoscopy were performed at diagnosis and during follow-up. Endoscopic severity in UC patients was assessed according to the Mayo subscore for endoscopy [17]. Endoscopic severity in CD patients was assessed by the Simple Endoscopic Score for CD (SES-CD) [19,20]. 

### 2.4. Endpoints

The primary endpoints included the following: (1) induction of remission (defined as a Mayo score ≤2 in UC patients and an HBI ≤ 5 in CD patients) in patients treated with GP2017 compared with ADA originator; and (2) safety (defined as the absence of adverse events [AE] during treatment) of GP2017 and ADA originator.

AEs occurring at the injection time were classified as early, while AEs occurring at least 1 week after the injection were defined late events. AEs were graded as mild (not required to stop treatment) or severe (requirement to stop treatment). The occurrence of opportunistic infections was also regarded as an AE [21]. Opportunistic infections are those due to microorganisms that in normal circumstances have limited pathogenic capacity but able to induce a disease because of the predisposing effect of another concomitant disease or its treatment [22].

The secondary endpoints assessed differences between GP2017 and ADA originator in terms of: (1) clinical response (defined as a decrease of at least 2 points in the Mayo score in UC patients and at least 3 points in the HBI in CD patients); (2) mucosal healing (defined as a Mayo subscore for endoscopy ≤ 1 in UC and SES-CD ≤ 2 in CD patients); (3) reduction of steroid use during the study period; (4) prevention of colectomy in UC and any disease-related surgical procedure in CD; and (5) optimization rate to reach remission for the biosimilar GP2017 during follow-up. To exclude any bias on mucosal healing evaluation in patients switched from ADA originator to biosimilar, we evaluated mucosal healing only in patients naive to GP2017 who had performed colonoscopy. Therapeutic optimization was allowed as follows: 40 mg every week or 80 mg every 2 weeks for both the ADA originator and biosimilar GP2017.

### 2.5. Statistical Analysis

Data analysis was perfomed with MedCalc® Release 14.8.1. 

Continuous non-parametric variables were reported as median (interquartile range [IQR]), and categorical variables were reported as number (percentage). The Shapiro-Francia test was used to test for normal distribution. The Chi-square test was used to compare categorical variables, and the Mann–Whitney test was used to compare continuous variables. *p*-values of <0.05 were considered to be significant.

## 3. Results

A total of 134 patients (median age 38 [IQR 28–53] years; 50% male) were enrolled according to the inclusion criteria. Forty-one patients were diagnosed with UC and 93 patients with CD. Patients with UC had a median disease duration of 7 years (IQR 4–11), while for patients with CD, the median disease duration was 5 years (IQR 2–10). Thirty-five (85.4%) patients with UC and 83 (89.2%) patients with CD were naïve to ADA. 

For the total population, 62 (46.3%) patients received GP2017 while 72 (53.7%) patients received ADA originator. Significantly more patients with CD than UC had undergone a previous appendectomy (*p* < 0.007). For both UC and CD, the main indication to use ADA biosimilar was “steroid dependency”; “steroid resistance” was the second most common indication in patients with UC, while this was “others” for patients with CD. Median FC levels were significantly higher in patients with UC than CD (335 versus 134 µg/g; *p* = 0.033).

The characteristics of the study group are reported in Table 1.

### 3.1. Primary Endpoint

Patients’ median follow-up time was of 12 (IQR 6–12) months. 

Overall, 105 of 134 (78.4%) patients reached clinical remission, with no significant difference between treatment groups. Specifically, clinical remission was obtained in 51 of 62 (82.3%) patients treated with GP2017 and 54 of 72 (75.0%) patients treated with ADA originator (*p* = 0.311). 

In patients naïve to biologics clinical remission was obtained by 93 of 118 (78.8%) patients, including 39 of 46 (84.8%) in the GP2017 group and 54 of 72 (75.0%) in the ADA originator group (*p* = 0.207). 

For the 16 patients naïve to ADA but previously exposed to other TNFα inhibitors or anti-integrin, 12 (75.0%) patients reached clinical remission, all from the GP2017 group. 

### 3.2. Adverse Events

Only 2 (4.9%) patients with UC and 3 (3.2%) with CD experienced an AE (Table 2). Treatment discontinuation following AEs was reported for two patients treated with ADA originator and 1 patient treated with GP2017, with no significant difference between treatment groups. 

No cases of malignancy, tuberculosis, or death were reported during the study. 

### 3.3. Secondary Endpoints

The outcomes of secondary endpoints are reported in Table 3. Both clinical response and steroid reduction were similarly high in the two treatment groups, with no significant difference observed (*p* = 0.692 and *p* = 0.910, respectively). Mucosal healing was obtained by a significantly higher proportion of patients after therapy with GP2017 than ADA originator (89.2% versus 60.2%, respectively; *p* = 0.003). There were no surgeries performed in either treatment group during follow-up.

Optimization was required by 9 (6.7%) patients in total; 7 (11.3%) patients in the GP2017 group and 2 (2.8%) in the ADA originator group, with a trend toward difference (*p* = 0.051).

## 4. Discussion

Biosimilar drugs are gaining momentum as they offer accessible and cost-effective treatment alternatives for various autoimmune disorders, including IBD [23], while reference biologics approach patent expiry. Biosimilars are ‘similar’ but not identical to the ‘reference’ biologic, hence extrapolating the therapeutic indications and interchangeability with the originators remains a practical concern [6].

The US Food and Drug Administration and the European Medicines Agency approved adalimumab biosimilar GP2017 in 2018 [24,25]. The efficacy and safety of GP2017 has been established in multicenter, randomized, clinical trials in psoriasis [26] and rheumatoid arthritis [27]. GP2017 was approved for use in patients with IBD through extrapolation of indications. To date one study has determined that four ADA biosimilars, including GP2017, were efficacious and safe in treating IBD outpatients, comprising both biologic-naïve patients and those switched from the ADA originator, in a real-life Italian setting [11]. Hence, there is a paucity of data comparing the efficacy and safety of GP2017 with its originator ADA in IBD patients.

The results of our study show that biosimilar GP2017 is as effective and safe as its ADA originator in the IBD patient population, in both naϊve patients and those previously treated with TNFα inhibitors. Specifically, clinical remission was obtained in approximately 80% of patients overall, with no significant difference between GP2017 and ADA originator. Furthermore, 82.3% of patients treated with GP2017 obtained clinical remission, which agrees with data obtained in 81.8% (9 of 11) of IBD patients treated with GP2017 in a separate real-life Italian study [11].

Importantly, the biologic status of patients did not affect clinical remission in our study. In ADA-naïve patients, who represented almost 90% of the study population, clinical remission was obtained in more than 75% of patients with no significant difference in terms of biological treatment. Interestingly, GP2017 was also effective in most patients with prior exposure to TNFα inhibitors, although this patient population was limited in size.

Our study also found that GP2017 was similar to its ADA originator in clinical response and steroid reduction. Interestingly, the rate of mucosal healing was significantly higher in GP2017-treated patients than in the ADA originator group: it could be hypothesized that patients who switched from the ADA originator to GP2017 were already in remission. However, the optimization rates show that more patients treated with GP2017 switched to the weekly dose than those receiving ADA originator. To explain these results, we hypothesize that patients already treated with ADA would still need an optimization rate. On the other hand, we cannot exclude a “nocebo” effect that could negatively influence the performance of the ADA biosimilar [28], requiring more frequent optimization. 

Finally, GP2017 seems to be as safe as the ADA originator in real-life clinical practice, with only five AEs reported, and no between-group significance for AE-related treatment discontinuation.

Our study has strengths and limitations. One strength is the multicentric organization, which allow collection of data from several Gastroenterology Units, making the study population representative of a range of geographical areas across Italy thus limiting single center clinical bias. However, the main limitation is the retrospective design which did not allow the definition “a priori” of the patient number to power the analysis, leading to the small sample size. In addition, retrospective design did not allow enrolled patients to have the same timing throughout follow-up (both as clinical and endoscopic follow-up). Moreover, the follow-up period was limited to 12 months and we recognize that a longer follow-up would add insightful information. Nevertheless, it should be acknowledged that ADA biosimilar GP2017 was approved in Italy in January 2019 and had only been in clinical practice for approximately 20 months at the time of our study. The ongoing nature of our study will allow for a longer follow-up period in future analyses.

## 5. Conclusions

This observational study is the first analysis to show that the ADA biosimilar GP2017 is as safe and effective as its originator ADA in managing IBD patients in real-life clinical practice in Italian IBD centers. This results strongly support a wider use of ADA biosimilars in the routine clinical practice also for IBD patients. This would reduce the total costs for the hospitals without affecting therapy efficacy and patient safety. To confirm these promising results, randomized, double-blind, prospective studies with higher patient numbers and a longer follow-up are necessary.

## Figures and Tables

**Table 1:** Patient demographics, disease characteristics, and concomitant medications.

	Total (134 pts)	UC (41 pts)	CD (93 pts)	*p*-Value
Sex, male	67 (50.0)	16 (39.0)	51 (54.8)	0.093
Median (IQR) age in years (range)	38 (28–53)	47 (29–54)	39 (26–50)	0.129
Median (IQR) disease duration in years (range)	7 (3–15)	7 (4–11)	5 (2–10)	0.099
Body mass index, kg/m^2^ (range)	23 (22–24)	22 (21–24)	23 (21–25)	0.371
Presence of comorbidities	31 (23.1)	9 (22.0)	23 (23.7)	0.830
Smoke	42 (31.3)	9 (22.0)	33 (35.5)	0.121
Previous appendectomy	25 (18.7)	2 (4.9)	23 (24.7)	**<0.007**
Concomitant therapy				
- Mesalazine	83 (61.9)	40 (97.6)	43 (46.2)	
- Steroids	76 (56.7)	31 (75.6)	45 (48.4)	**<0.000**
- Tiopurine	21 (15.7)	3 (7.3)	18 (19.4)	
Indication to therapy with anti-TNFα				
- Steroid dependency	76 (56.7)	30 (73.2)	46 (49.5)	**0.005**
- Steroid resistance	25 (18.7)	9 (21.9)	16 (17.2)	
- Switch	8 (6.0)	-	8 (8.6)	
- Others	25 (18.7)	2 (4.9)	23 (24.7)	
Previous anti-TNFα	16 (11.9)	6 (14.6)	10 (10.8)	0.525
Naïve to ADA	118 (88.1)	35 (85.4)	83 (89.2)	0.534
Therapy				
- GP2017	62 (46.3)	20 (48.8)	42 (45.2)	
- ADA originator	72 (53.7)	21 (51.2)	51 (54.8)	0.699
Montreal classification of extent of UC				
- Proctitis		2 (4.9)		
- Left-sided colitis		16 (39.0)	-	
- Extensive colitis		23 (56.1)	-	
Montreal classification of CD				
- Disease location				
- Isolated ileal disease		-	47 (50.5)	
- Isolated colonic disease		-	12 (12.9)	
- Ileocolonic disease		-	33 (35.5)	
- Isolated UGI disease		-	1 (1.1)	
- Concomitant perianal disease		-	42 (45.2)	
- Disease behaviour				
- Nonstricturing, nonpenetrating		-	57 (61.3)	
- Stricturing		-	28 (30.1)	
- Penetrating		-	8 (8.6)	
Median (IQR) CRP in mg/L (range)	3 (2–5)	2.8 (2–6)	3.0 (3–4)	0.148
Median (IQR) fecal calprotectin in µg/g (range)	229 (89–560)	335 (212–582)	134 (78–207)	**0.033**
Median (IQR) partial Mayo score (range)		8 (6–10)	-	
Median (IQR) Mayo subscore for endoscopy (range)		2 (2–3)	-	
Median (IRQ) HBI (range)		-	2 (1–4)	
Median (IRQ) SES-CD (range)		-	5 (1–8)	

**Data are given as number (percentage) of patients unless otherwise indicated.** ADA, Adalimumab; CD, Crohn’s disease; IQR, interquartile range; CRP, C-reactive protein; GP2017, biosimilar to ADA originator; HBI, Harvey-Bradshaw index; SES-CD, simple endoscopic score for Crohn’s disease; TNFα, tumor necrosis factor α; UC, Ulcerative colitis; UGI, upper gastrointestinal.

**Table 2:** Frequency of adverse events.

		UC				CD		
	Total (41 pts)	ADA Originator (21 pts)	GP2017 (20 pts)	*p*-Value	Total (93 pts)	ADA Originator (51 pts)	GP2017 (42 pts)	*p*-Value
**Total AEs**	**2 (4.9)**	**2 (9.5)**	**0 (0)**	**ns**	**3 (3.2)**	**2 (3.9)**	**1 (2.4)**	**ns**
**Mild-moderate AEs**	**1 (2.4)**	**1 (4.8)**	**0 (0)**	**ns**	**1 (1.1)**	**1 (2.0)**	**0 (0)**	**ns**
- Allergy	-	-	-	-	1 (1.1)	1 (2.0)	0 (0)	ns
- Headache	1 (2.4)	1 (4.8)	0 (0)	ns	-	-	-	-
**Severe AEs**	**1 (2.4)**	**1 (4.8)**	**0 (0)**	**ns**	**2 (2.2)**	**1 (2.0)**	**1 (2.4)**	**ns**
- Allergy	1 (2.4)	1 (4.8)	0 (0)	ns	1 (1.1)	0 (0)	1 (2.4)	ns
- Rectal abscess	-	-	-	-	1 (1.1)	1 (2.0)	0 (0)	ns

**Data are given as number (percentage) of patients.** AE, Adverse events; ADA, Adalimumab; CD, Crohn’s disease; GP2017, biosimilar to ADA originator; UC, Ulcerative colitis.

**Table 3:** Outcomes of secondary end-points during follow-up.

	Total (134 pts)	GP2017 (62 pts)	ADA Originator (72 pts)	*p*-Value
Clinical response ^a^	115 (85.8)	54 (87.1)	61 (84.7)	0.692
Mucosal healing ^b^	63/87 * (72.4)	33/37 (89.2)	30/50 (60.0)	**0.003**
Reduction of steroids ^c^	125 (93.3)	58 (93.5)	67 (93.1)	0.910
Optimization ^d^	9 (6.7)	7 (11.3)	2 (2.8)	

**Data are given as number (percentage) of patients unless otherwise indicated.** GP2017, biosimilar to ADA originator. ^a^ decrease of at least 2 points in the Mayo score in UC patients and at least 3 points in the HBI in CD patients. ^b^ Mayo subscore for endoscopy ≤ 1 in UC and SES-CD ≤ 2 in CD patients. ^c^ steroids use during the study period. ^d^ 40 mg every week or 80 mg every 2 weeks for both the ADA originator and GP2017. * Mucosal healing was assessed only in a subset of patients.

## Data Availability

The data presented in this study are available on request from the corresponding author.

## References

[1] GBD 2017 Inflammatory Bowel Disease Collaborators (2020). The global, regional, and national burden of inflammatory bowel disease in 195 countries and territories, 1990–2017: A systematic analysis for the Global Burden of Disease Study 2017. Lancet Gastroenterol. Hepatol..

[2] Ng S.C., Shi H.Y., Hamidi N., Underwood F.E., Tang W., Benchimol E.I., Panaccione R., Ghosh S., Wu J.C.Y., Chan F.K.L. (2017). Worldwide incidence and prevalence of inflammatory bowel disease in the 21st century: A systematic review of population-based studies. Lancet.

[3] Lamb C.A., Kennedy N.A., Raine T., Hendy P.A., Smith P.J., Limdi J.K., Hayee B., Lomer M.C.E., Parkes G.C., Selinger C. (2019). British Society of Gastroenterology consensus guidelines on the management of inflammatory bowel disease in adults. Gut.

[4] Blackstone E.A., Fuhr J.P. (2012). Innovation and Competition: Will Biosimilars Succeed?: The creation of an FDA approval pathway for biosimilars is complex and fraught with hazard. Yes, innovation and market competition are at stake. But so are efficacy and patient safety. Biotechnol. Healthc..

[5] Norum J., Koldingsnes W., Aanes T., Antonsen M.A., Florholmen J., Kondo M. (2011). The economic burden of TNFalpha inhibitors and other biologic treatments in Norway. Clinicoecon. Outcomes Res..

[6] Danese S., Fiorino G., Raine T., Ferrante M., Kemp K., Kierkus J., Lakatos P.L., Mantzaris G., van der Woude J., Panes J. (2017). ECCO Position Statement on the Use of Biosimilars for Inflammatory Bowel Disease—An Update. J. Crohn’s Colitis.

[7] Tursi A., Mocci G., Lorenzetti R., Allegretta L., Brandimarte G., Cassieri C., Colucci R., De Medici A., Faggiani R., Ferronato A. (2021). Long-term real-life efficacy and safety of infliximab and adalimumab in the treatment of inflammatory bowel diseases outpatients. Eur. J. Gastroenterol. Hepatol..

[8] Cingolani L., Barberio B., Zingone F., Ferronato A., Bertani L., Costa F., Bodini G., Demarzo M.G., Melatti P., Gubbiotti A. (2021). Adalimumab biosimilars, ABP501 and SB5, are equally effective and safe as adalimumab originator. Sci. Rep..

[9] Ribaldone D.G., Caviglia G.P., Pellicano R., Vernero M., Saracco G.M., Morino M., Astegiano M. (2020). Effectiveness and safety of adalimumab biosimilar ABP 501 in Crohn’s disease: An observational study. Rev. Esp. Enferm. Dig..

[10] Tapete G., Bertani L., Pieraccini A., Lynch E.N., Giannotta M., Morganti R., Biviano I., Naldini S., Mumolo M.G., De Nigris F. (2022). Effectiveness and Safety of Nonmedical Switch from Adalimumab Originator to SB5 Biosimilar in Patients with Inflammatory Bowel Diseases: Twelve-Month Follow-Up from the TABLET Registry. Inflamm. Bowel Dis..

[11] Tursi A., Mocci G., Allegretta L., Aragona G., Bianco M.A., Colucci R., Cuomo A., Della Valle N., Ferronato A., Forti G. (2022). Comparison of Performances of Adalimumab Biosimilars SB5, APB501, GP2017, and MSB11022 in Treating Patients with Inflammatory Bowel Diseases: A Real-Life, Multicenter, Observational Study. Inflamm. Bowel Dis..

[12] Agenzia Italiana del Farmaco (Italian Medicines Agency) Regime di Rimborsabilità e Prezzo di Vendita del Medicinale per uso Umano «Hyrimoz». (Determina n. 72/2019). Gazzetta Ufficiale della Repubblica Italiana 2019;34:1-4 (Reimbursement Regime and Selling Price of the Medicinal Product for Human Use "Hyrimoz". [Resolution no. 72/2019]. [19A00797] [GU General Series n.34 of 09-02-2019]). https://www.gazzettaufficiale.it/eli/id/2019/02/09/19A00797/sg.

[13] Ministero della Salute, Attività dei Comitati Etici Istituiti ai Sensi del Decreto Ministeriale 18 Marzo 1998 (2002). CIRCOLARE MINISTERIALE N. 6 DEL 2 SETTEMBRE 2002. G.U. n. 214 12 settembre 2002. https://www.aosp.bo.it/sites/default/files/2002-09-02_0.pdf.

[14] DM 18.03.1998 Linee guida di Riferimento per L’istituzione e il Funzionamento dei Comitati etici (2022). GU n. 122 del 28 maggio 1998, 1998. https://bit.ly/3oks6rq.

[15] Provvedimento 31 Luglio 2002 (2002). GU n. 230 1/10/2002. https://bit.ly/3z2NrvG.

[16] Satsangi J., Silverberg M.S., Vermeire S., Colombel J.F. (2006). The Montreal classification of inflammatory bowel disease: Controversies, consensus, and implications. Gut.

[17] Schroeder K.W., Tremaine W.J., Ilstrup D.M. (1987). Coated oral 5-aminosalicylic acid therapy for mildly to moderately active ulcerative colitis. A randomized study. N. Engl. J. Med..

[18] Best W.R. (2006). Predicting the Crohn’s disease activity index from the Harvey-Bradshaw Index. Inflamm. Bowel Dis..

[19] Daperno M., D’Haens G., Van Assche G., Baert F., Bulois P., Maunoury V., Sostegni R., Rocca R., Pera A., Gevers A. (2004). Development and validation of a new, simplified endoscopic activity score for Crohn’s disease: The SES-CD. Gastrointest. Endosc..

[20] Moskovitz D.N., Daperno M., Assche G.A. (2007). Defining and validating cut-offs for the Simple Endocopic Score for Crohn’s Disease. Gastroenterology.

[21] Roda G., Jharap B., Neeraj N., Colombel J.F. (2016). Loss of Response to Anti-TNFs: Definition, Epidemiology, and Management. Clin. Transl. Gastroenterol..

[22] Colombel J.F., Sandborn W.J., Reinisch W., Mantzaris G.J., Kornbluth A., Rachmilewitz D., Lichtiger S., D’Haens G., Diamond R.H., Broussard D.L. (2010). Infliximab, azathioprine, or combination therapy for Crohn’s disease. N. Engl. J. Med..

[23] Fiorino G., Gilardi D., Correale C., Furfaro F., Roda G., Loy L., Argollo M., Allocca M., Peyrin-Biroulet L., Danese S. (2019). Biosimilars of adalimumab: The upcoming challenge in IBD. Expert Opin. Biol. Ther..

[24] Generics and Biosimilars Initiative (GaBI) Biosimilars approved in Europe. https://www.gabionline.net/biosimilars/general/biosimilars-approved-in-europe.

[25] Generics and Biosimilars Initiative (GaBI) Biosimilars approved in the US. https://www.gabionline.net/biosimilars/general/Biosimilars-approved-in-the-US.

[26] Blauvelt A., Lacour J.P., Fowler J.F., Weinberg J.M., Gospodinov D., Schuck E., Jauch-Lembach J., Balfour A., Leonardi C.L. (2018). Phase III randomized study of the proposed adalimumab biosimilar GP2017 in psoriasis: Impact of multiple switches. Br. J. Dermatol..

[27] Wiland P., Jeka S., Dokoupilová E., Brandt-Jürgens J., Miranda Limón J.M., Cantalejo Moreira M., Cabello R.V., Jauch-Lembach J., Thakur A., Haliduola H. (2020). Switching to Biosimilar SDZ-ADL in Patients with Moderate-to-Severe Active Rheumatoid Arthritis: 48-Week Efficacy, Safety and Immunogenicity Results from the Phase III, Randomized, Double-Blind ADMYRA Study. BioDrugs Clin. Immunother. Biopharm. Gene Ther..

[28] D’Amico F., Solitano V., Peyrin-Biroulet L., Danese S. (2021). Nocebo effect and biosimilars in inflammatory bowel diseases: What’s new and what’s next?. Expert Opin. Biol. Ther..

